# Effect of CXCR2 Inhibition on Behavioral Outcomes and Pathology in Rat Model of Neuromyelitis Optica

**DOI:** 10.1155/2018/9034695

**Published:** 2018-12-13

**Authors:** Melina V. Jones, Michael Levy

**Affiliations:** Department of Neurology, Johns Hopkins University, Baltimore, MD, USA

## Abstract

**Objective:**

To reduce immune-mediated damage in a rat model of neuromyelitis optica (NMO) by blocking neutrophil migration using SCH527123, a drug that inhibits CXCR2.

**Background:**

Neuromyelitis optica is a relapsing autoimmune disease that preferentially targets the optic nerves and spinal cord leading to blindness and paralysis. Part of the immunopathogenesis of this disease is thought to involve neutrophils, which are present within NMO lesions. We tested the effect of blocking neutrophil migration in an NMO rat model.

**Methods:**

The Lewis rat model of NMO uses a myelin-reactive experimental autoimmune encephalomyelitis (EAE) background with passive transfer of pooled human antibody from patients with aquaporin-4 (AQP4) seropositive NMO at onset of EAE symptoms. We treated rats early in the course of EAE with CXCR2 inhibitor and assessed the extent of neutrophil infiltration into the spinal cord and the extent of AQP4 depletion.

**Results:**

CXCR2 inhibitor decreased neutrophil migration into the spinal cord of AQP4 IgG-treated EAE rats. However, there was no difference in the acute behavioral signs of EAE or the extent and distribution of AQP4 lesions. This suggests that neutrophils are not centrally involved in the immunopathogenesis of the Lewis rat NMO disease model.

**Conclusions:**

CXCR2 inhibitor blocks neutrophil migration into the spinal cord during EAE but does not significantly reduce inflammation or AQP4 lesions in the Lewis rat model of NMO.

## 1. Background

Neuromyelitis optica spectrum disorder (NMOSD) is a devastating autoimmune disease that targets the optic nerves and spinal cord leading to blindness and paralysis [1]. The pathology of NMOSD is distinguished from other demyelinating diseases, such as multiple sclerosis, by the presence of the aquaporin-4 (AQP4) serological antibody and by humorally mediated inflammatory markers associated with AQP4-depleted lesions, including perivascular immunoglobulin and complement [2, 3]. In addition, neutrophils and other granulocytes are present in lesions in the spinal cord and optic nerve where they have been speculated to contribute to the permanent destruction of the myelin, glial cells, and vulnerable neurons [4].

Neutrophils have been identified as a key player in the effector pathway of NMO disease and are being targeted for intervention in clinical trials [5, 6]. Animal models of NMO demonstrated that neutrophils are important in lesion development; inhibition of neutrophils reduces the severity and size of lesions [7]. CXCR2, also known as IL8RB, is a receptor for interleukin-8, CXCL1, and GRO-beta, which are secreted by neutrophils as a chemoattractant to amplify neutrophil recruitment to sites of injury and inflammation [8]. We tested the potential benefit of inhibiting neutrophil recruitment with a small molecular CXCR2 inhibitor, SCH527123, in a rat model of NMO. The purpose was to evaluate the impact of CXCR2 inhibition on the severity of immune-mediated damage in the central nervous system.

## 2. Methods

### 2.1. Rats and Induction of EAE

All animal studies were approved by the Johns Hopkins Animal Care and Use Committee. 8-week-old female Lewis rats (~150 g; Envigo, Indianapolis, USA) were housed in the pathogen-free animal facility at Johns Hopkins on a 12-hour day light cycle. Complete Freund's adjuvant containing 10 mg/ml heat-killed Mycobacterium tuberculosis H37RA (Difco) in Incomplete Freund's adjuvant (Imject; Thermo Scientific) was mixed 1 : 1 with a 1 mg/ml solution of guinea pig myelin basic protein (gpMBP, Sigma USA, or extracted from guinea pig spinal cord) in PBS and mechanically emulsified for 10 minutes (Norm-Ject luer lock; cat. no. 4100-X00V0; Thermo, MX5341L). EAE was induced by subcutaneous injection of 100 *μ*l of this mixture with a 20-gauge needle at the base of the tail under isoflurane anesthesia (2.5% Forane in oxygen). All rats were weighed and scored daily starting at day 5 postimmunization. Scoring was assigned using a standard 5 point scale: 0, no deficit; 0.5, partial loss of tail tone or slightly abnormal gait; 1.0, complete tail paralysis or both partial loss of tail tone and mild hind limb weakness; 1.5, complete tail paralysis and mild hind limb weakness; 2.0, tail paralysis with moderate hind limb weakness (evidenced by frequent foot dragging or turning over while walking); 2.5, no weight-bearing on hind limbs (dragging) but with some leg movement; 3.0, complete hind limb paralysis with no residual movement; 3.5, hind limb paralysis with mild weakness in forelimbs; 4.0, complete quadriplegia but with some movement of the head; 4.5, moribund; and 5.0, dead. Animals reaching a score of 3.5 for >1 day or a score of 4 at any time were euthanized. Water source is low to the floor of the cage such that paralyzed animals can easily reach it; food was placed on the floor of the cage when signs of any paralysis were noted.

### 2.2. NMO-IgG Preparation

AQP4 antibody (NMO-IgG) was among the total IgG from plasmapheresate donated by aquaporin-4 antibody-seropositive donors undergoing treatment for a relapse; fluids were stored at −80°C until use. Plasmapheresate was diluted 3-fold in PBS, clarified at 10,000 ×g for 15 minutes at 4°C, and filtered with 70 *μ*m strainer (Millipore) to remove particulate materials. Clarified solution was loaded on to 8 ml columns of Protein G-Sepharose (Biovision Inc., cat. no. 6511-25) equilibrated with phosphate buffered saline (PBS). Columns were then washed with 10 column volumes (CV) of PBS. Purified IgG was eluted with 0.1 M glycine-HCl, pH 2.6 into 1.5 ml tubes containing 90 *μ*l of neutralization solution (1 M Tris-HCl pH 9.0). Fractions with 280 nm absorbance greater than 5-fold over background were pooled and buffer-exchanged to PBS using Amicon spin filters (50 KDa MWCO, 15 ml; Millipore). The final volume was adjusted to 7–10 ml of PBS and sterile filtered. The final yield typically was 140–160 mg of IgG from 50 ml of plasmapheresate.

### 2.3. AQP4 IgG/Drug Treatment of Lewis Rats

**Figure 1:**
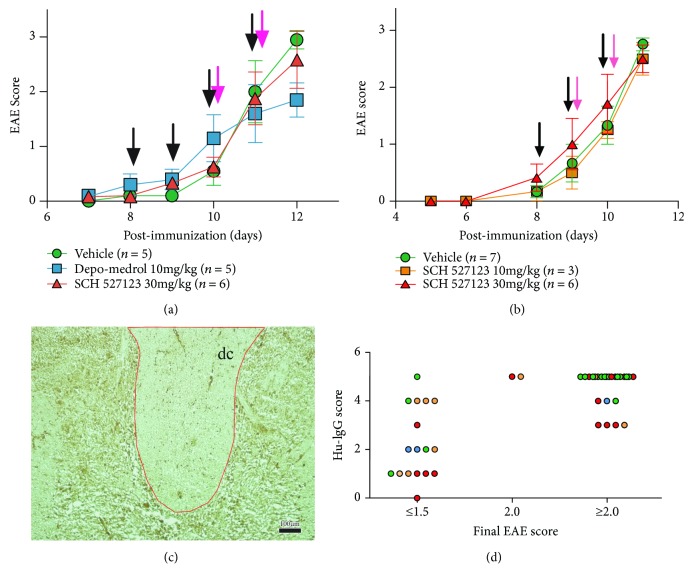
(a, b) After immunizing Lewis rats with gpMBP to induce EAE, CXCR2 antagonist (red triangles) or vehicle control (green circles) was injected at the time points indicated by the black arrows and pooled IgG from AQP4-seropositive NMO patients was injected at the time points indicated by the pink arrows. Treatment with corticosteroid, Depo-Medrol, on days 8 and 10 was included in cohort 1 ((a) blue squares), while cohort 2 tested SCH527123 at both 10 mg/kg (orange squares) and 30 mg/kg (red triangles). EAE scores were not exacerbated by IgG from AQP4-seropositive NMO patients. (c) Diffusion of human IgG (Hu-IgG) into the spinal cord was assessed by immunohistochemistry (brown stain) and scored semiquantitatively. (d) Human IgG (Hu-IgG) was associated with higher EAE scores, while lower EAE scores were associated with lower levels of human IgG signal in the spinal cord.

Vehicle (0.5% hydroxypropyl methylcellulose) or CXCR2 antagonist, SCH527123 drug (generously provided by Genzyme), was injected as indicated in Figures 1(a) and 1(b), intraperitoneally, at 10 or 30 mg/kg on 3-4 days preceding tissue collection. Pooled purified IgG from three AQP4 seropositive patients was injected intraperitoneally on the two days preceding tissue collection. Depending on the cohort, each animal received 12 mg (cohort 1) or 15 mg (cohort 2) of IgG on each of the two days. Rats were euthanized the day following the last injection. One cohort included a control treatment arm with methylprednisolone acetate on days 8 and 10 postimmunization (Depo-Medrol, 10 mg/kg, subcutaneous, Pfizer).

### 2.4. Tissue Collection, Embedding, and Sectioning

Rats were terminally anesthetized by inhalation of isoflurane via necrosis chamber (VWR) and perfused with 75 ml of cold 100 mM HEPES, pH 7.3, 5 mM sodium pyruvate, 2 mM KCl, 2 mM MgCl2, and 124 mM NaCl through the left cardiac ventricle. Following decapitation, the unfixed spinal cord was immediately removed, placed lengthwise on a flat wooden stick, and frozen by immersing in a 500 ml bath of dry ice-cooled 2-methylbutane and stored at −80°C until OCT embedding (Sakura Tissue-Tek). Ten micron sections of tissue from 6–8 spinal levels were placed on Fisherbrand Plus slides and immediately placed in a dry ice-cooled slide box. Slides were stored at −80°C until used.

### 2.5. Immunohistochemistry

Slides were removed from −80°C and air-dried for 5 minutes. A hydrophobic barrier (Liquid blocker Super PAP pen; Electron Microscopy Sciences, USA) was applied around sections either before (paraformaldehyde) or after (acetone) fixation. Fixation was performed by immersion in either freshly prepared ice cold 4% (w/v) paraformaldehyde (PFA) in PBS, pH 7.4, for 1 hour (T cells, C4d, microglia, human IgG, GFAP) or 20 minutes (His48; neutrophils), or in acetone (C5b-9 membrane attack complex, C7, C9) for 10 minutes at −10°C. PFA-fixed slides were then washed in PBS while acetone-fixed slides were air-dried for 5 minutes then washed in Tris-buffered saline (TBS).

Staining conditions are summarized in Table 1. Alkaline phosphatase procedures used TBS for washes and diluents and signal was developed with 5-bromo-4-chloro-3-indolyl phosphate (4-toluidine salt) with 4-nitro blue tetrazolium chloride (BCIP+NBT; Sigma-Roche) using 0.1 M Tris-HCl pH 9.5, 0.1 M NaCl, 5 mM MgCl2 supplemented with 4 mM tetramisole hydrochloride as a development buffer and levamisole to block endogenous phosphatases (Sigma). Horseradish peroxidase procedures (ABC-HRP) used PBS + 0.1% Triton®-X100 (SigmaUltra) for washes and diluents; endogenous peroxidases were quenched by immersing slides in 3% H_2_O_2_ in PBS for 20 minutes and washed before blocking with 5% goat serum. When ABC-HRP was used, endogenous biotin reactivity was blocked by supplementing the 5% goat serum (Sigma) during blocking with 1 : 20 avidin and the primary antibody staining solution with 1 : 20 biotin (Avidin-biotin blocking kit; Vector Labs), with 3 × 2 min washes with PBS in between. All primary antibody incubations were performed overnight at 4°C except MAC, C9 (90 minutes, room temperature), and anti-human-IgG (30 minutes, room temperature). Anti-rabbit Immpress Alkaline Phosphatase (Vector Labs) was used without dilution (C4d), while alkaline phosphatase-conjugated anti-mouse IgM (Sigma) used for His48 staining was diluted in 5% goat serum in TBS with 0.1% Triton-X100. Staining with anti-C5-C9 omitted all detergents and used 2% (w/v) bovine serum albumin (Fraction V, Sigma A9056; use of serum in this procedure was extremely detrimental to staining).

Biotinylated antibodies were all diluted 1 : 1000 (Vector Labs) and incubated on slides for 30 minutes followed by washing and incubation with Avidin-Biotin HRP complex (ABC, Vector Labs) for a further 30 minutes. HRP signal was developed by immersing slides in freshly prepared and filtered 3,3′-diaminobenzidine tetrahydrochloride hydrate (Sigma) for 5 minutes (0.05% in PBS with 0.03% H_2_O_2_). Peroxidase-reacted slides were dehydrated, Fast Green-counterstained, and mounted as previously described [9]. Some BCIP+NBT-reacted slides were counterstained with 0.01% Ponceau S in 0.5% acetic acid (Sigma), washed with 50 mM Na acetate pH 5.3, and mounted without coverslip with In Situ Mounting Medium (Electron Microscopy Sciences, cat. no. 17988-30).

### 2.6. Quantitative Immunohistochemical Analysis

Human IgG signal was scored on a semiquantitative scale of 0 (no antibody signal) to 5 (complete and intense coverage of all spinal cord segments examined for a given animal) in a blinded manner. Other immunoreactive markers were quantified as described [9]. Briefly, high-resolution pictures of each spinal cord segment were acquired at 4× and background corrected. ImageProPlus5.1 (Media Cybernetics, Rockville, MD, USA) was employed to determine the total area of each tissue section (8–10 axial spinal sections per rat) and the total immunoreactive area, with the same color choices for quantification applied to all slides stained in the same batch. Data points are expressed as total % Immunoreactive Area (total stained area/total area × 100%), and the mean ± standard error of the mean are represented by a horizontal line with *y*-error bars.

### 2.7. Statistics

Student's *t*-test was performed on immunohistochemistry results. A result of *p* < 0.05 was considered significant. Results are presented via tools and analysis using GraphPad Prism software.

## 3. Results

### 3.1. Study Design

Lewis rats immunized with gpMBP to induce EAE and then injected with pooled IgG from AQP4-seropositive patients developed AQP4-depleted lesions in the spinal cord. The injection of human IgG is most pathological in the first 24–48 hours from behavioral onset of disease. To examine neutrophil involvement in lesion development, a CXCR2 small molecule antagonist, SCH527123, or vehicle control (0.5% methylcellulose) was injected into EAE rats starting on day 8 postimmunization and on the days of IgG injections (black arrows, Figures 1(a) and 1(b)). We tested the hypothesis that treatment with SCH527123 would prevent the influx of neutrophils into the spinal cord and would subsequently attenuate the formation of AQP4-depleted lesions.

### 3.2. Behavioral Outcomes

Injections of IgG from NMO patients did not significantly exacerbate EAE scores within the short time period examined in this experiment (Figure 1(a)). In addition, treatment with SCH527123 at either 10 mg/kg or 30 mg/kg did not affect the development of EAE behavioral disease (Figure 1(b)). Conversely, corticosteroid (Depo-Medrol) treatment before injection with IgG significantly attenuated EAE development relative to the vehicle control (*p* < 0.02 on day 12 postimmunization). Despite the limited use of this NMO rat model for evaluation of neurological deficits due to AQP4 IgG, the model is useful to study the pathological mechanisms of astrocyte damage due to AQP4 IgG and complement.

### 3.3. Pathological Outcomes at the Blood-Brain Barrier

Behavioral signs of EAE progressed quickly once initial signs were observed, often progressing from a score of 1.0 (limp tail) to 3.0 (complete hind limb paralysis) in a span of 24 hours beginning at day 10 or 11 postimmunization. At the onset of neurological disability, blood-brain barrier (BBB) integrity is briefly compromised allowing injected human IgG into the CNS (Figures 1(c) and 1(d)), along with other serum proteins and any drugs that had been injected that might not enter the CNS under basal conditions. In contrast, animals injected with human IgG before behavioral disease onset had moderate to no detectible human IgG in the CNS (Figure 1(d)). Immunohistochemical signal for human antibody in the spinal cord was most robust in animals that have a score of 1.0 to 2.0 on the day of injection of pooled IgG from AQP4 seropositive patients and have a score of ≥2.5 on the following day.

### 3.4. Pathological Outcomes in the Spinal Cord

**Figure 2:**
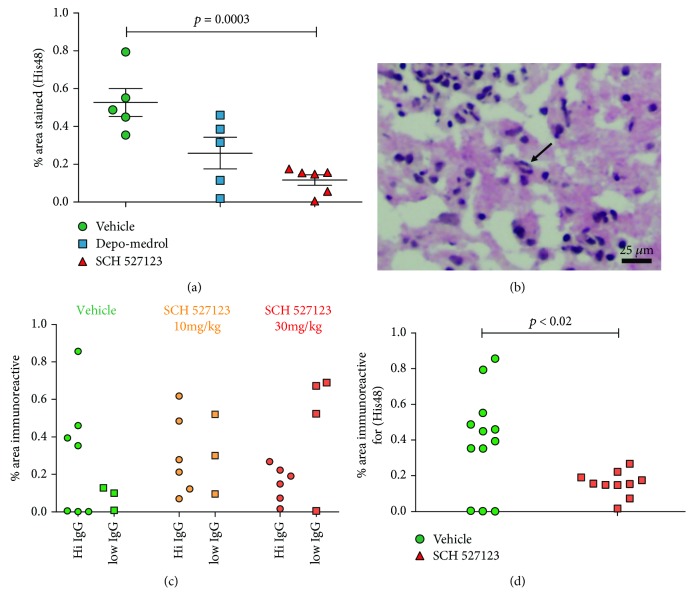
SCH527123 decreases neutrophil migration into the spinal cord of EAE rats with high human IgG levels. (a) In this cohort, Hu-IgG levels were high in both the vehicle- and SCH527123-treated animals. At 30 mg/kg, SCH527123 treatment (red triangles) significantly decreased neutrophil accumulation in the spinal cord of EAE rats when compared to the vehicle-treated group (green circles, *p* < 0.0003). (b) Examples of His48+neutrophils in the spinal cord of EAE rats (arrows, bar = 25 *μ*m). (c) In this cohort, where Human IgG levels in the CNS were more variable, there is a trend towards decreased neutrophil recruitment in SCH527123 treatment at 30 mg/kg compared to vehicle-treated animals only in animals with high Hu-IgG scores, but high variability in the vehicle-treated group limits the differences between these groups (Student's *t*-test *p* = 0.362; variance is significantly increased *p* < 0.02). (d) Pooling results for rats with high IgG in their spinal cords from the two cohorts for vehicle and SCH527123 treatment at 30 mg/kg indicate that this higher dose significantly decreases His48+neutrophil recruitment following high levels of human IgG deposition in the spinal cord of EAE rats (*p* < 0.02).

In the first cohort, neutrophil accumulation in the CNS was significantly decreased in animals treated with SCH527123 compared to the vehicle-treated group (Figure 2(a); *p* < 0.0005). Corticosteroid (Depo-Medrol) treatment also decreased neutrophil influx (*p* < 0.05). In a second cohort which compared 10 and 30 mg/kg of SCH527123, the higher dose of drug in animals with high IgG levels showed a trend towards blocking neutrophil influx compared to the vehicle-treated group. The degree of variability in the vehicle-treated group was significantly increased compared to the SCH527123-treated group (Figure 2(c), *F* ratio: *p* < 0.02). When responses from animals with high IgG deposits in the CNS from both cohorts are combined, the overall result demonstrates that treatment with SCH527123 at 30 mg/kg decreased the influx of neutrophils induced by NMO-IgG by 60% (Figure 2(d), *p* < 0.02).

**Figure 3:**
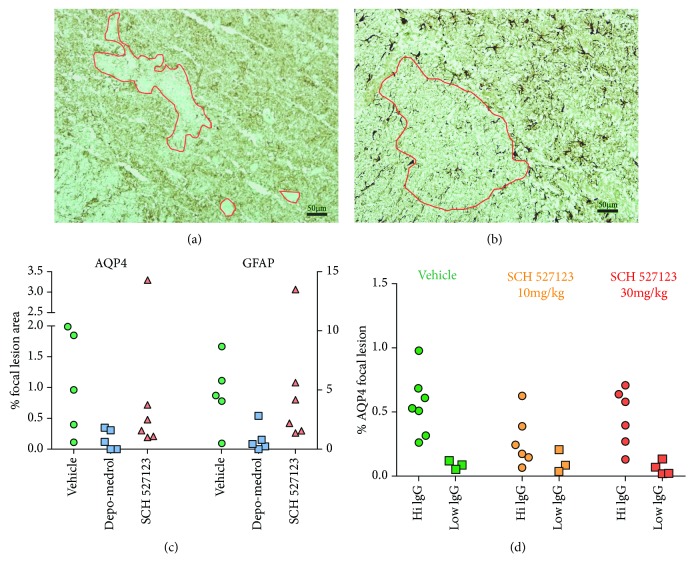
Astrocyte damage is not attenuated by treatment with CXCR2 antagonist, SCH527123. Examples of focal astrocyte death demonstrated by loss of AQP4 (brown stain (a)) or GFAP (brown stain (b)) immunohistochemistry in adjacent tissue sections (bar = 50 *μ*m). In the first cohort (c), AQP4- and GFAP-depleted lesions (left and right *y*-axis, respectively) were not significantly decreased by SCH527123 treatment at 30 mg/kg (red triangles) compared to vehicle-treated rats (green circles). Depo-Medrol treatment at 10 mg/kg (blue squares) significantly decreased lesion load (*p* = 0.042). In the second cohort (d), there was no significant astrocytic protective effect of SCH527123, even when the groups were subdivided by IgG deposition, where the “hi IgG” animals showed relatively higher levels of IgG deposition and AQP4/GFAP loss compared to the “low IgG” animals.

Pooled IgG from AQP4-seropositive NMO patients historically causes focal areas of Aqp4 loss in this rat EAE model.  Figure 3(a) shows an example of a typical AQP4-depleted lesions in this rat EAE model, and Figure 3(b) shows an adjacent section stained for GFAP (glial fibrillary acidic protein). We quantified AQP4-depleted lesions as a percentage of total spinal tissue area in the sections examined and found that SCH527123 does not reduce the lesion area compared to vehicle control (Figure 3(c)). As expected, corticosteroids do have a protective effect. In rats that showed only low deposits of human IgG, AQP4-depleted lesions did not develop, regardless of control or treatment group (Figure 3(d)).

**Figure 4:**
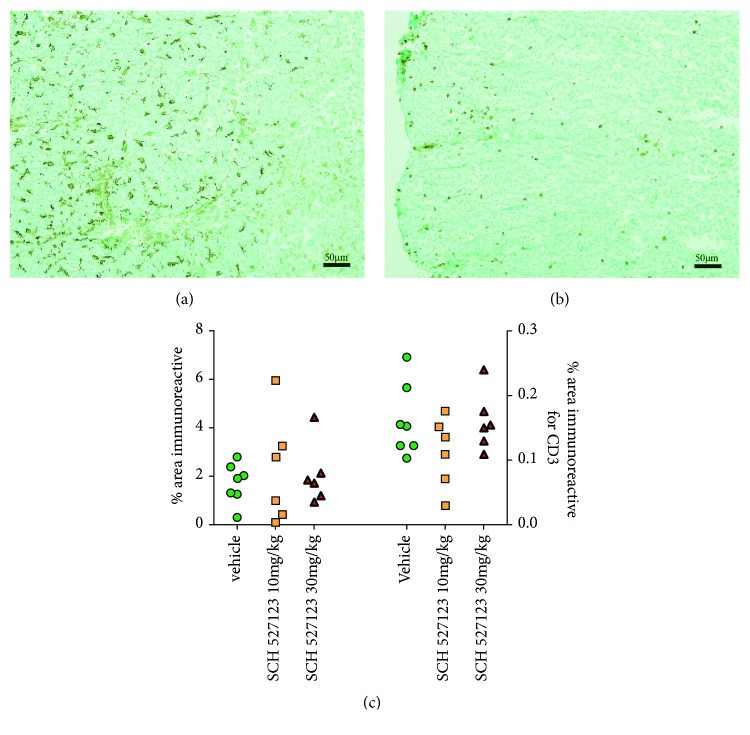
CXCR2 antagonist, SCH527123, does not alter macrophage/microglial activation or CD3 T cell influx into the spinal cord. Representative sections of immunohistochemistry using IBA1 to mark macrophages and microglia are shown (a) and CD3 to mark T cells (b) (bar = 50 *μ*m). Focusing only on animals in cohort 2 for which Hu-IgG signal and astrocyte damage were high, neither IBA1 (left axis) nor CD3 (right axis) signal was altered by SCH527123 treatment (blue squares, 10 mg/kg; red triangles, 30 mg/kg) as compared to vehicle-treated animals (green circles).

**Figure 5:**
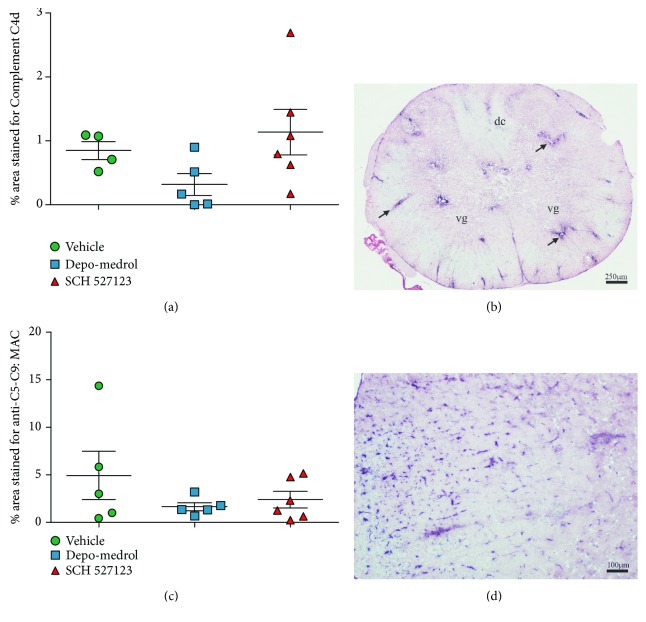
Complement markers are unchanged in NMO rats treated with CXCR2 antagonist, SCH527123. C4d, a marker for activation of the classical complement pathway, showed similar staining patterns in and around spinal cord lesions from rats treated with SCH527123 compared to vehicle control, quantified (a) with a representative sample (b). Depo-Medrol at 10 mg/kg showed lower levels of C4d staining. Staining with anti-C5-C9 was used to assess overall terminal activation of complement pathways. C5-C9 was not significantly different among the three groups, quantified (c) with a representative sample (d).

Treatment with SCH527123 did not significantly alter the levels of activated macrophage/microglial lineage cells in the spinal cords of EAE rats, as measured by the marker IBA-1 (Figures 4(a) and 4(c)). In addition, despite reports that a subset of T cells expresses CXCR2, numbers of infiltrating T cells (CD3+) were also not significantly affected by SCH527123 treatment (Figures 4(b) and 4(c)). Complement markers were also not significantly reduced by SCH527123 treatment including classical complement marker C4d (Figures 5(a) and 5(b)) or terminal complement markers C5b-9 (Figures 5(c) and 5(d)).

## 4. Discussion

The Lewis rat model of NMO is a useful animal model in which to test the role of neutrophils in the pathology of immune-mediated damage in NMO [10]. This particular rat model is carried out in a Lewis rat on a background of experimental autoimmune encephalomyelitis (EAE) using T cells reactive to myelin basic protein, in this case, generated by direct immunization. Pooled immunoglobulin from NMO patients who are seropositive for the aquaporin-4 antibody is then passively transferred into rats early in the course of EAE. In this model, the aquaporin-4 (AQP4) antibody specifically binds astrocytes in the rat central nervous system leading to antibody-mediated astrocytopathy, consistent with the human disease pathology [11, 12].

The complement activation pathway, initiated by human antibody fixation of rat complement, generates C5a, which attracts neutrophils [13]. Both CXCR2 ligands and C5a alone are neutrophil chemoattractants. Although the mechanistic link between C5a and CXCR2 ligands is poorly understood, in our complement-driven model, it appears that blocking CXCR2 with the small molecule CXCR2 antagonist, SCH527123, decreases neutrophil migration into the CNS. However, the prevention of neutrophil migration into the CNS did not result in the reduction of AQP4 lesions, suggesting that neutrophils are not central to this part of the pathogenesis of NMO. Close physical association of CXCR2 and C5a receptors (C5aR) on the surface of neutrophils has been reported [14]. Both receptors use similar intracellular signaling pathways involving Gi protein-mediated induction of protein kinase C, biphasic intracellular calcium increases, and of activation of ERK1 and 2 [15, 16]. CXCR2 is known to form heterodimers with other receptors, which can positively or negatively regulate each partner's response to its ligands and antagonists [17, 18]. It has been reported that C5a treatment of human neutrophils interferes with calcium responses to subsequent CXCR2 ligand treatment but that CXCR2 ligand does not block C5a responses [14]. SCH527123 is thought to lock CXCR2 into a form that is not conducive to signaling but allows ligand to bind (a so-called “allosteric inverse agonist” [19]); if CXCR2 and C5aR form heterodimers on neutrophils, our results suggest that SCH527123 may block responses to both neutrophil chemoattractants (CXCR2 ligands and C5a) while only interfering with the signaling to the receptor for one (CXCR2).

Behaviorally, there was no apparent benefit for rats that received SCH527123. However, this model is not ideal for assessing this outcome measure. Our rats did not show significant neurological worsening with passive transfer of the AQP4 IgG. Other investigators have shown that AQP4 IgG exacerbates behavioral scores of EAE rats [20, 21]. We speculate that the difference in outcomes may be due to varying toxicity of AQP4 IgG clones used by other investigators [22–24]. Alternatively, we may have terminated the study too early to see this effect [21].

Neutrophils are primarily thought of as a first-line defense against invading pathogens, using a combination of Fc- and complement receptor-driven pathways to pour toxic granule contents and oxygen-derived free radicals on to target cells [25, 26]. Neutrophils also invade tissues in a wide variety of injury models and they can have both positive and negative roles in repair, depending on the tissue and the injury. For example, neutrophils have been implicated with impeding functional recovery from spinal cord injury by promoting glial scarring in the CNS [27] but also have been shown to promote repair in the peripheral nervous system through phagocytic functions [28]. Neutrophils and neutrophil expression of CXCR2 are essential to the development of EAE [29, 30] and in the progression of cuprizone-induced demyelination [31]. While reducing neutrophil accumulation up to 60% with SCH527123 treatment did not significantly protect astrocytes from damage in our study, it is possible that low levels of neutrophil involvement were sufficient to cooperate with the classical complement pathway to initiate damage.

This study used a CXCR2 antagonist to inhibit neutrophil recruitment to the CNS. Many cell types have been reported to express CXCR2 constitutively or after induction (reviewed by [32]), including subsets of T cells, macrophages, and endothelial cells [33]. In CXCR2 null mice, the predominant phenotype is on neutrophil migration. Additional studies confirmed that while CXCR2 function on monocytes responds to activation by migration inhibitory factor (MIF), monocyte migration can function without CXCR2, likely due to the compensation by other chemokine receptors including CCR2, CX3CR1, and CCRs1-5. Other than neutrophils, we did not observe reduction of migration of any other cell type in this Lewis rat NMO model. CXCR2 is also expressed in nonhematopoietic cells that may have an impact on CNS demyelinating models. For example, CXCR2 is expressed on oligodendrocyte precursor cells where it limits remyelination by interfering with their expansion and migration [34]. Oligodendrocyte precursor cells within demyelinated lesions of CXCR2 null mice proliferated earlier and more vigorously [34]. CXCR2 also represents a viable target for promoting remyelination following traumatic injury, dysmyelinating conditions, or infection [35–38].

There are two significant limitations to the Lewis rat model of NMO. First, the disease course is initiated by myelin-reactive T cells, which is not AQP4 specific. It should be noted, however, that in this Lewis rat EAE model, MBP-reactive T cells (whether generated by direct immunization or by adoptive transfer of MBP-reactive T cell lines) do not mediate any cell damage without the subsequent passive transfer of pathogenic autoantibodies. Second, the AQP4 specificity depends on human IgG fractions from patients with NMO. While mouse monoclonal antibodies directed against rat AQP4 transferred into EAE rats induced large confluent AQP4-depleted lesions, the passive transfer of human IgG into EAE rats causes relatively much smaller lesions not reproducible between human IgG specimens. This may be due to a lower affinity of the human antibody for the rat AQP4 [12]. Alternatively, this may be due to specimen-specific variation in the mode of toxicity of the antibodies [22], which might influence our ability to block astrocyte damage by targeting only neutrophils.

In conclusion, impeding neutrophil migration into the CNS by CXCR2 antagonism was successful but failed to protect AQP4 from damage by passively transferred human IgG from NMO patients. CXCR2 antagonism may have benefits beyond neutrophil involvement for the treatment of autoimmune-mediated CNS disease and should be explored further.

## Figures and Tables

**Table 1:** Immunohistochemistry markers.

Marker	Working dilution (clone)	Source company	Fixation/time	Detection	Chromogen
Human IgG	1 : 1000	Vector Labs	PFA, 1 h	Elite ABC-HRP (Vector Labs)	DAB
Neutrophils	1 : 250 (His48)	BD Pharmingen	PFA, 15 min	Alk. phos. anti-ms IgM (Sigma)	BCIP+NBT
Aquaporin-4	1 : 250 (H-80)	Santa Cruz Biotechnology	PFA, 1 h	Elite ABC-HRP	DAB
GFAP	1 : 250	Dako USA	PFA, 1 h	Elite ABC-HRP	DAB
Macrophage/microglia (rabbit anti-iba1)	1 : 1500 (IHC)	Wako Lab. Chem. USA	PFA, 1 h	Elite ABC-HRP	DAB
T cells (CD3)	1 : 60 (sp7)	GeneTex, Inc.	PFA, 1 h	Elite ABC-HRP	DAB
C4d	1 : 1000	American Research Products Inc.	PFA, 1 h	Immpress anti-rabbit alk. phos. (Vector Labs)	BCIP+NBT
Anti-C5-C9	1 : 1000 (#204903)	Calbiochem	Acetone, 10 min	Immpress anti-rabbit alk. phos.	BCIP+NBT

## Data Availability

The data used to support the findings of this study are included within the article.
